# Long-term performance of seagrass restoration projects in Florida, USA

**DOI:** 10.1038/s41598-019-51856-9

**Published:** 2019-10-29

**Authors:** Ryan J. Rezek, Bradley T. Furman, Robin P. Jung, Margaret O. Hall, Susan S. Bell

**Affiliations:** 1 Department of Integrative Biology, University of South Florida, 4202 E Fowler Ave, Tampa, FL 33620 USA; 2 Fish and Wildlife Research Institute, Florida Fish and Wildlife Conservation Commission, 100 8th Ave SE, St., Petersburg, FL 33701 USA

**Keywords:** Restoration ecology, Marine biology, Ecology, Ecology, Ocean sciences

## Abstract

Seagrass restoration is a common tool for ecosystem service enhancement and compensatory mitigation for habitat loss. However, little is known about the long-term performance of these projects. We identified seagrass restoration projects by reviewing historic permitting documents, monitoring reports, and studies conducted in Florida, USA, most of which have not been cited previously in peer-reviewed literature. We then revisited 33 seagrass restorations ranging in age from 3 to 32 years to compare seagrass percent cover, species diversity, and community structure in restored and contemporary reference seagrass beds. We found that 88% of restoration projects continued to support seagrass and, overall, restored percent cover values were 37% lower than references. Community composition and seagrass percent cover differed from references in projects categorized as sediment modification and transplant restorations, whereas all vessel damage repair projects achieved reference condition. Seagrass diversity was similar between restored and reference beds, except for sediment modification projects, for which diversity was significantly lower than in reference beds. Results indicate that restored seagrass beds in Florida, once established, often exhibit long-term persistence. Our study highlights the benefit of identifying and surveying historic restorations to address knowledge gaps related to the performance and long-term fate of restored seagrass beds.

## Introduction

Seagrass meadows provide a variety of critical ecosystem services, including fisheries enhancement^1–3^, coastal erosion reduction^4,5^, and water quality improvement^6^, and have undergone extensive damage in many areas. Seagrass habitat loss has been linked to anthropogenic disturbances, including nutrient loading, coastal development, and vessel impacts^7–9^. Presently, seagrass meadows are among the most threatened ecosystems on earth, having lost an estimated 29% of areal cover since 1879^9^. The true status of seagrass meadows, however, is not well known^10^, given that the majority of seagrasses are found in subtidal areas where damage can go unseen and/or monitoring programs have not yet been established.

Seagrass restoration has become a common management tool for recovering the ecological functions and services lost due to habitat fragmentation and degradation^11–13^. Yet, recent estimates have ranked seagrass beds among the most expensive coastal habitats to restore^14^, and the success of restoration projects can be difficult to predict. The latter is evident from systematic reviews of seagrass restoration that have highlighted the uncertainty of successful outcomes. In 1998, 50% of seagrass restoration projects in the United States failed to meet stated success criteria^11^, and a more recent study reported global seagrass survival rates of 37%^13^ among restoration trials. The comparatively low rate of restoration success has been attributed to a variety of factors including inappropriate site selection (e.g., poor sediment or hydrological conditions), natural environmental perturbations, insufficient planting efforts, and/or ongoing human stressors^11,13,15^.

An important goal for any restoration effort is that the ecosystem becomes resilient to normal ranges of environmental stress and has the capacity to persist in the absence of ongoing human intervention^16^. However, this remains difficult to assess as most studies on seagrass restoration undergo a short monitoring period, with a median duration of 1–1.5 years^11,17^. Some studies have monitored the performance of restorations for 2 to 3 years post-planting^18–21^, but very few have extended the evaluation beyond 5 years^17,22,23^, calling into question the long-term (i.e., >5 years) effectiveness of seagrass restoration as a management tool. Despite appeals to expand monitoring periods and to document long-term restoration performance^14,15,17,19,24,25^, the availability of long-term monitoring data from past seagrass restorations remain limited.

Seagrass mitigation, i.e., restoration activities undertaken to compensate for damage to seagrass beds, is generally required in the USA as a condition for the issuance of permits for public and private development projects that destroy seagrasses, in compliance with the “no net loss” wetlands policy outlined in Section 404 of the Clean Water Act^26^. Seagrass mitigation efforts are especially common in the subtropical coastal waters of Florida, USA, where extensive and widely distributed seagrass meadows have been subjected to impacts arising from human population growth and coastal urbanization^27^. As a result, a substantial number of mitigation projects have been conducted in Florida, although the data contained in permit documentation and monitoring have been largely overlooked in the peer-reviewed literature, with the reports themselves scattered among numerous state and federal agencies. Lack of accessibility is disconcerting because reporting data represent the accumulated knowledge of practitioners and researchers with respect to design, implementation, and monitoring of seagrass restoration projects. Its absence from the primary literature, therefore, risks redundant experimental work and avoidable negative outcomes^15^.

Given the common use of seagrass restoration to offset anthropogenic habitat degradation, it is important to gain a comprehensive understanding of the long-term performance of these projects to evaluate their effectiveness as a tool for ecosystem management and advance the field of coastal restoration ecology. In this study, we identified seagrass restoration projects by reviewing historic permitting documents, monitoring reports, and studies conducted in Florida, most of which have not been cited previously in peer-reviewed literature. We then revisited and surveyed historic projects that were originally deemed successful, or were trending towards success, to evaluate seagrass abundance and community composition in comparison to contemporary natural references. Using this approach, we evaluated the long-term status of restoration projects, identified the effectiveness of different restoration strategies, and characterized current structural attributes of restored seagrass communities.

## Results

### Seagrass cover

**Figure 1:**
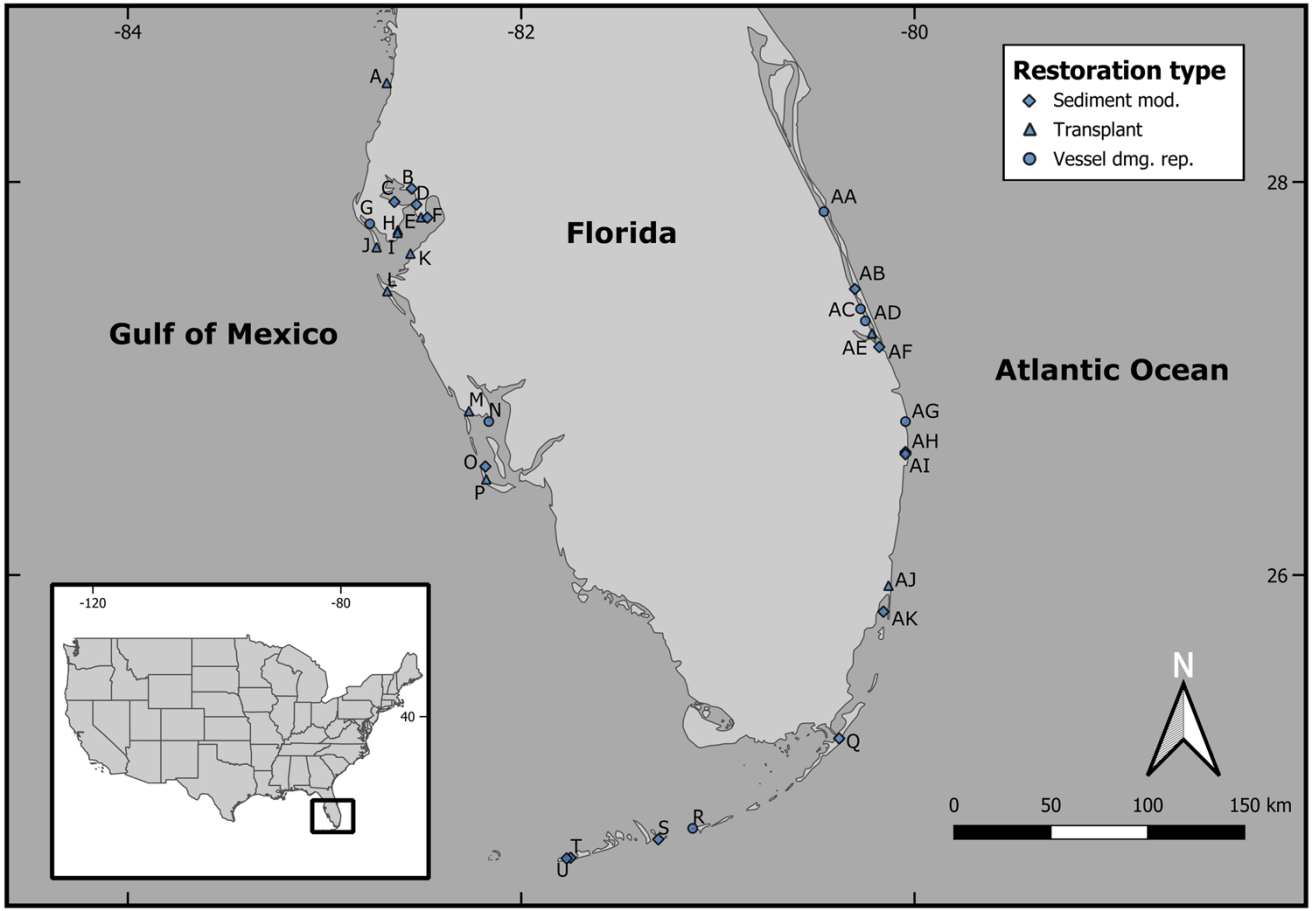
Map of seagrass restoration sites visited in this study, with restoration type (sediment modification, transplant, vessel damage repair) indicated. Map generated using QGIS (version 3.0.1, http://qgis.osgeo.org).

**Figure 2:**
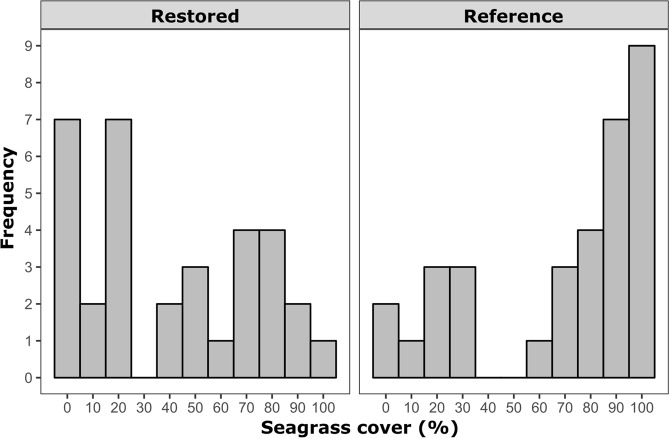
Mean percent cover of seagrass from restored and reference beds across sites (10% increments; *n* = 33).

**Figure 3:**
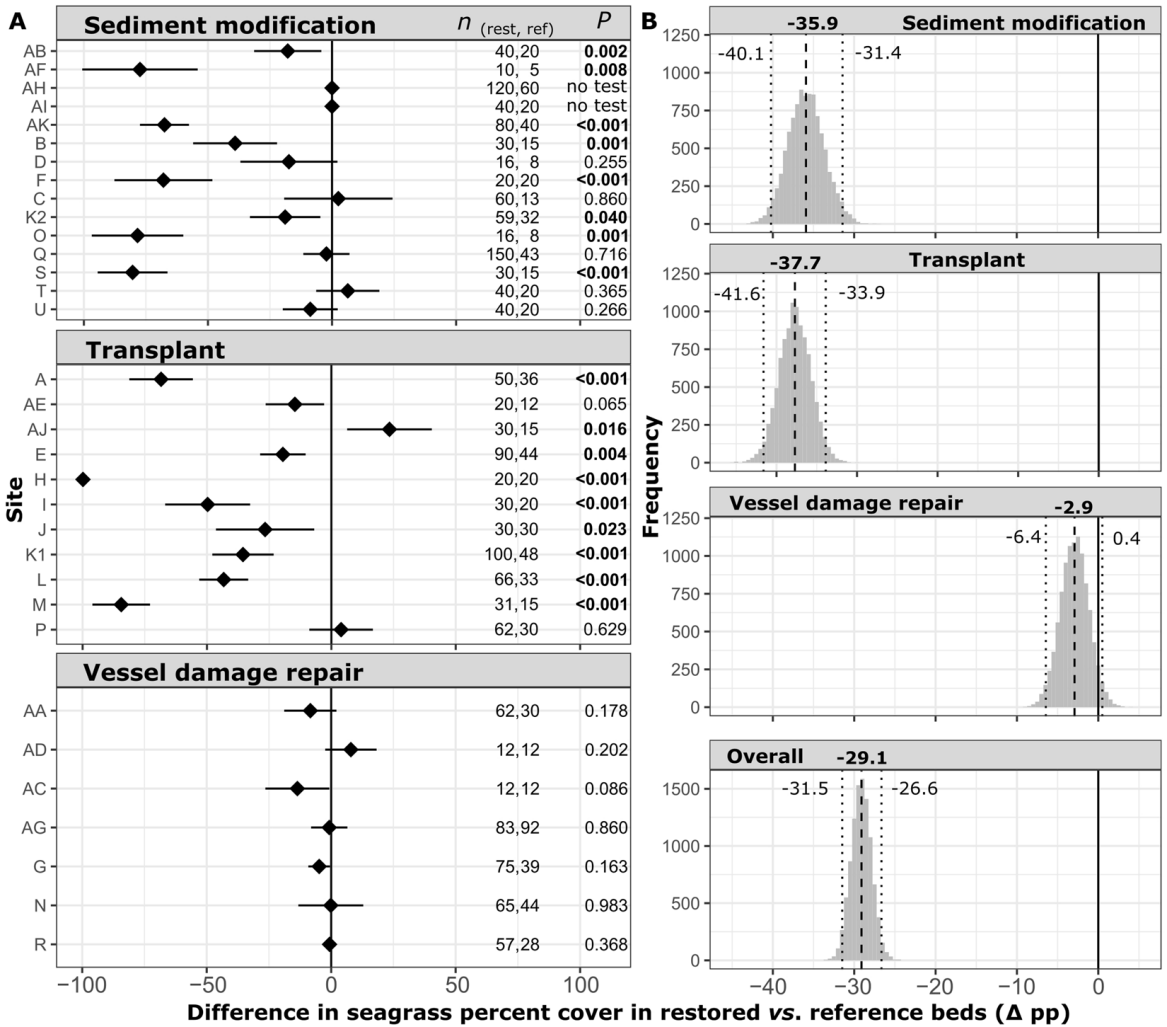
Mean and 95% confidence interval differences of seagrass percent cover between restored and reference bed in each site (Δµ = µ_Restored_ − µ_Reference_). Values represent percentage points (pp), with sample size and adjusted permutation test *P*-values (α = 0.05; bold) annotated (no test = no seagrass in restoration or references) (**A**). Bootstrap distribution of overall and restoration type-specific mean differences in seagrass cover between restored and reference beds (0.5 increments) (**B**). Mean (bold) and 95% bootstrap confidence intervals are annotated; means with confidence intervals that do not include 0 are considered significantly different.

Thirty-three sites, composed of seagrass restoration projects and their respective natural references, were selected for sampling in Florida coastal waters, spanning latitudes from 24.5° to 28.5° N (Fig. 1; Table 1; Supplemental Table S1). Seagrass was found in 88% of restoration projects (29/33) sampled in this survey. Mean seagrass percent cover values ranged from 0 to 98.6% in restoration projects with a grand mean of 41.7 ± 5.8% (median = 35.6%); references ranged from 0 to 99.9% with a grand mean of 68.4 ± 5.9% (median = 84.5%) (Fig. 2; Supplemental Table S2). Among sites comparatively evaluated for seagrass abundance (*n* = 31), 52% (*n* = 16) of restorations had significantly lower percent cover than their respective references, and 1 site (3%) had significantly greater cover in restored beds (Fig. 3A). When compared by restoration type, seagrass percent cover in restored beds was significantly lower than reference beds in 62% (8/13) of sediment modification and in 73% (8/11) of transplant sites. No vessel damage repair projects differed from references in terms of percent cover (0/7).

**Table 1:** Restoration project data indicating the restoration type (SM = sediment modification; TP = transplant; VD = vessel damage repair), habitat modifications (PU = seagrass planting units; SF = sediment fill; SR = sediment removal; BW = breakwater; ST = sediment tubes; VR = vessel removal), purpose of project (M = mitigation, R = habitat restoration), year of site visit, the age of the restoration when sampled, and sampled area of restoration.

Site ID	Rest. type	Hab. Mod.	Purpose	Year sampled	Age at visit (yr)	Area (ha)
A	TP	PU	M	2018	8	2.841
AA	VD	ST	M	2016	8	0.283
AB	SM	SF, PU	M	2016	12	0.769
AC	VD	VR	M	2016	5	0.008
AD	VD	PU	M	2016	8	0.004
AE	TP	PU	M	2016	10	0.015
AF	SM	SR	M	2016	7	0.058
AG	VD	VR	M	2016	7	0.085
AH	SM	FL	M	2016	13	18.211
AI	SM	FL	M	2018	4	1.837
AJ	TP	PU	M	2016	9	0.105
AK	SM	SF, PU	M	2018	3	6.718
B	SM	SF, PU	M	2016	10	0.405
C	SM	SF	M	2016	8	1.267
D	SM	SR	M	2016	13	0.113
E	TP	PU	R	2018	12,9,8^a^	0.360
F	SM	BW^b^	R	2018	8	2.882
G	VD	ST	M	2016	7	0.032
H	TP	BW, PU	M	2018	15	0.016
I	TP	PU	R	2018	31	0.060
J	TP	PU	R	2018	16	0.608
K1	TP	PU, BW	M	2018	18	7.236
K2	SM	SR, PU	M	2018	18	8.555
L	TP	PU	M	2016	13	0.469
M	TP	PU	M	2016	8	0.008
N	VD	ST, PU	M	2016	9	0.016
O	SM	SF	R	2016	7	0.037
P	TP	PU	M	2016	8	0.445
Q	SM	SR, PU	M	2016	32	5.666
R	VD	ST	R	2016	8	0.364
S	SM	SR, PU	M	2016	13	0.162
T	SM	SF, PU	M	2018	6	1.056
U	SM	SF, PU	M	2018	18	1.052

^a^Restoration took place in 3 phases over time.

^b^Breakwater construction only, considered SM because the goal was to accrete sediment.

When all sites were considered, mean seagrass percent cover was 37.2 ± 7.6% lower in restored seagrass beds than respective reference beds, with a mean difference of −29.1 pp (95% CI = −31.5, −26.6) (Fig. 3B). When compared by restoration type, percent cover in restored beds was found to be 46.8 ± 11.3% lower than reference beds in sediment modification sites (mean difference [95% CI] = −35.9 pp [−40.1, −31.4]), 40.6 ± 13.6% lower than reference beds in transplant sites (mean difference [95% CI] = −37.7 pp [−41.1, −33.7]), and statistically indistinguishable from reference beds in vessel damage repair sites (Fig. 3B).

### Seagrass diversity

**Figure 4:**
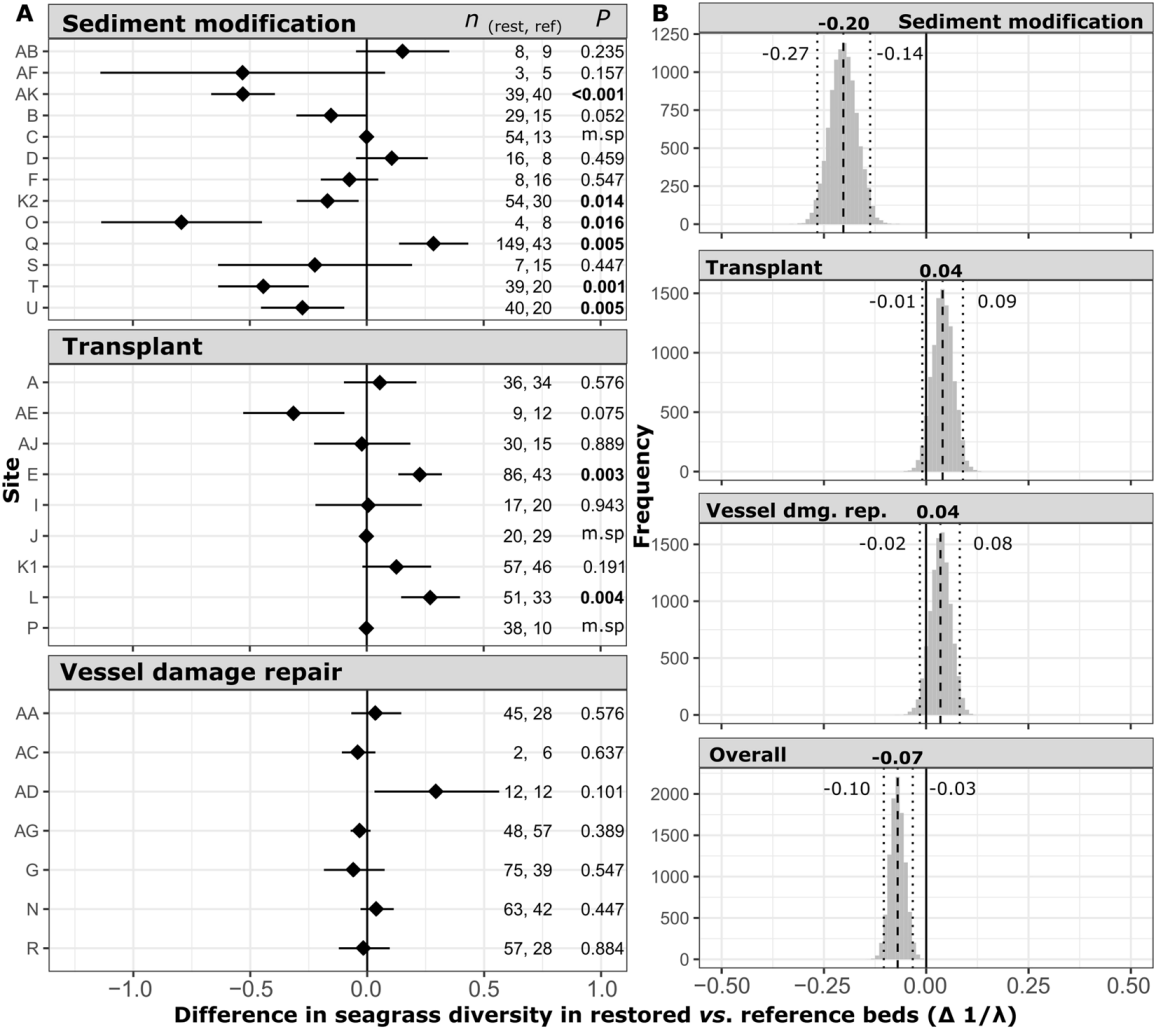
Mean and 95% confidence intervals of differences between restored and reference seagrass bed values for inverse Simpson diversity index (1/λ) within each restoration site (Δµ = µ_Restored_ − µ_Reference_), with sample size and adjusted permutation test *P*-values (α = 0.05; bold) annotated (tests were not conducted for monospecific sites = m.sp) (**A**). Bootstrap distribution of overall and type-specific mean difference in species number per sample values between restored and reference beds (0.01 increments) (**B**). Mean (bold) and 95% bootstrap confidence intervals are annotated; means with confidence intervals that do not include 0 are considered significantly different.

Six seagrass species were identified in field surveys (Supplemental Table S3), with *Thalassia testudinum* and *Halodule wrightii* being the two most prevalent in both restored and reference beds. Among sites comparatively evaluated for seagrass diversity (*n* = 29), inverse Simpson diversity index (1/λ) ranged from 1.00 to 1.76 in restored beds (mean = 1.21 ± 0.04) and 1.00 to 2.05 in reference beds (mean = 1.28 ± 0.06). Seagrass diversity in restored beds was significantly lower than references in 17% (5/29) of sites and significantly greater in 10% of sites (3/29) (Fig. 4A). Seagrass diversity in restored beds was similar or greater than reference values in all transplant and vessel damage repair sites but significantly lower than reference beds in 38% (5/13) of sediment modification sites.

Seagrass diversity in restorations was 3.0 ± 3.2% lower than references on average, with a paired mean difference of −0.07 (95% CI = −0.10, −0.03) (Fig. 4B). This minor overall difference was due to seagrass diversity being 11.3 ± 5.3% lower in restored beds than reference beds in sediment modification sites (mean difference [95% CI] = −0.20 pp [−0.27, −0.14]), while diversity in transplant and vessel damage repair sites were not significantly different when examined independently (Fig. 4B).

### Seagrass community composition

**Figure 5:**
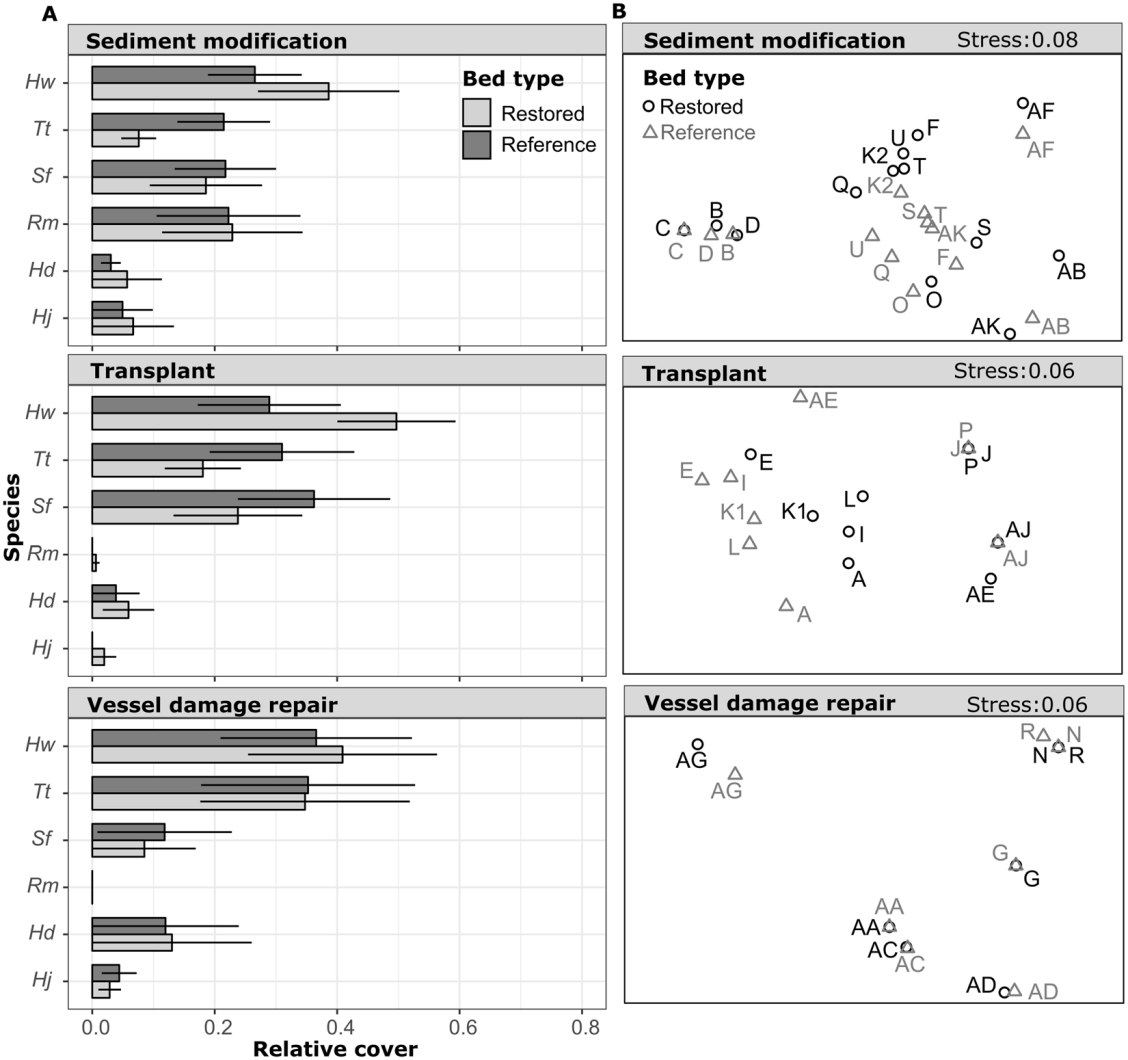
Mean ± SE of average cover values of seagrass species in restored and reference beds by site (**A**). Non-metric multidimensional scaling plots (nMDS) of Bray-Curtis dissimilarity between restored and reference beds based on site average cover data (square root transformed) (**B**). Species abbreviations: *Halodule wrightii* = *Hw*; *Thalassia testudinum* = *Tt*; *Syringodium filiforme* = *Sf*; *Ruppia maritima* = *Rm*; *Halophila decipiens* = *Hd*; *Halophila johnsonii* = *Hj*.

The results of a 2-way PERMANOVA on species relative abundance data indicated a significant interaction between bed type (restored/reference) and restoration type (Table 2). Post-hoc pairwise comparisons indicated that seagrass community composition differed between restored and reference beds in sediment modification (*P* < 0.01) and transplant (*P* < 0.01) sites (Table 2; Fig. 5A,B). Community composition in vessel damage repair projects did not differ from references (*P* = 0.59). Overall, seagrass community dissimilarity was primarily driven by a greater relative abundance of *H. wrightii* and a lower relative abundance of *T. testudinum* and *Syringodium filiforme* in restored beds in comparison to reference beds (Fig. 5A,B; Table 3). Differences in the relative abundance of these three species contributed to 77.8% of the cumulative compositional dissimilarity between restored and reference beds (Table 3).

**Table 2:** Results of two-way permutational multivariate analysis of variance on seagrass percent cover (square-root transformed) and pairwise comparisons of bed type (restored/reference) within restoration type (Type).

	*df*	SS	MS	*F*	R^2^	*P*
Bed	1	8.62	8.62	31.39	0.016	**0.0001**
Type	2	36.33	18.16	66.12	0.067	0.9999
Bed*Type	2	5.80	2.90	10.55	0.011	**0.0001**
Residuals	1786	490.66	0.28		0.906	
*Pairwise comp.*	*df*	SS	*MS*	^*F*^	*R* ^*2*^	*P*
**Sediment modification**						
Bed	1	5.48	5.48	4.46	0.028	**0.0001**
Residuals	690	190.11	0.28		0.97	
**Transplant**						
Bed	1	6.25	6.25	4.78	0.038	**0.0001**
Residuals	584	159.87	0.27		0.96	
**Vessel damage repair**						
Bed	1	1.47	1.47	2.32	0.010	0.5904
Residuals	512	140.68	0.27		0.99	

Significant *P*-values are in bold (α = 0.05).

**Table 3:** Results of SIMPER analysis of seagrass cover data between restored and reference beds.

Species	Contrib. to dissim. (%)	SD	Ave. Rest.	Ave. Ref.	Cum. sum. (%)
*H. wrightii*	21.3	19.0	0.433	0.287	30.5
*T. testudinum*	20.1	19.2	0.265	0.363	59.4
*S. filiforme*	12.8	19.0	0.125	0.188	77.8
*R. maritima*	7.0	16.0	0.106	0.050	87.7
*H. decipiens*	6.7	15.3	0.059	0.089	97.3
*H. johnsonii*	1.8	8.1	0.013	0.024	100.0

The average contribution to community dissimilarity, standard deviation, average cover in restored and reference beds, and cumulative contribution to dissimilarity are indicated for each seagrass species. Mean dissimilarity between restored and reference beds was 69.8%, overall.

### Influence of restoration age on recovery

Restoration age was not a significant factor in explaining mean differences in percent cover between restored and reference beds across sites for any restoration type (*P* > 0.05; Supplemental Fig. S1). Seagrass diversity was found to increase relative to references with restoration age in sediment modification projects (F_1,11 _ = 7.0, *P* = 0.02, R^2^ = 0.33; Supplemental Fig. S2). Restoration age did not explain the variation in seagrass diversity relative to reference controls for transplant or vessel damage repair projects (*P* > 0.05).

## Discussion

This study represents the largest and most comprehensive effort to revisit and evaluate historic seagrass restoration projects to date. By implementing standardized sampling methodologies and benchmarking restoration performance against references, we were able to characterize structural attributes of restored beds and evaluate the magnitude of difference in these attributes from natural equivalency. While previous literature-based studies provide a critical perspective on restoration outcomes over broad geographical ranges^11,13,14^, the ability of these analyses to describe the structure and performance of restored seagrass beds quantitatively has been limited by short-term monitoring durations, frequent lack of natural reference monitoring, and highly variable data formats provided in project reports. Our findings provide a unique perspective on seagrass restoration particularly with regard to community structure in restored seagrass beds and long-term restoration performance.

Seagrass restoration has frequently been viewed as an unpredictable management strategy, often with poor results^11,13–15^. A recent literature review conducted by van Katwijk *et al*.^13^ evaluated 1786 restoration trials and reported estimated seagrass restoration trial survival rates of 37% overall and 42% survival for large restorations (>100,000 shoots/seeds planted) after 22 months. The survival rate of individual planting units has also been estimated at 38% based on literature analysis^14^. These studies demonstrate that loss of seagrass in the early stages of projects remains a major hurdle in seagrass restoration. However, in contrast to the low initial survival rates previously reported, we observed long-term seagrass restoration persistence rates approaching 90%. Importantly, this study demonstrates that many of the seagrass restoration efforts initially deemed successful or trending toward success by Florida practitioners have created habitat capable of persisting for years to decades after management action. Our findings are promising, in that they demonstrate the long-term stability of seagrass restorations that survive beyond an initial establishment period and confirm the effectiveness of seagrass restoration as a tool for supporting the recovery of lost or degraded habitats. We conclude that seagrass restorations in Florida that persist unaided, do so as a result of sufficient planting efforts, appropriate site selection, and/or the use of adaptive management practices.

Seagrass abundance in restored beds varied substantially among projects and was generally sparser than reference beds, particularly within transplant and sediment modification sites. The absence of a temporal effect on seagrass percent cover suggests that adopting space for time substitution may be inappropriate at the timescales tested here (3–32 years after restoration) and/or that inter-annual variability in percent cover might be sufficiently high to mask decadal trends in restoration trajectories. Also, idiosyncratic aspects of project history, such as location, restoration methodology, and environmental fluctuations, likely result in substantial variation in project performance, potentially overriding age-associated trends in the recovery of seagrass abundance in this study; a larger sample set may be needed to further evaluate the role of age in recovery potential.

Development of seagrass cover is strongly influenced by physical processes, such as wind wave oscillation, current speed, and light availability, which all affect the growth and expansion of seagrasses and contribute to the characteristically heterogeneous structure of natural seagrass beds^28^. Seagrass abundance, as captured by visual estimates of percent cover, is also strongly influenced by relationships among complex self-reinforcing feedback mechanisms which can reduce the impacts of environmental fluctuations, or alternatively, limit seagrass recovery or lead to sudden unexpected shifts towards undesirable states^29,30^. Seagrass restorations may often fail to achieve the areal size or plant density necessary to activate self-sustaining positive feedback mechanisms necessary to advance recovery. Self-sustaining feedback mechanisms, such as the trapping of suspended sediments which further increases light penetration, sediment oxygenation which alleviates sulfide toxicity, and water current dampening effects of seagrass canopies which reduce physical stress, can strongly influence the recovery trajectory in seagrass beds (see Maxwell *et al*.^30^ for review). Focusing restoration efforts on large-scale plantings^13^ and implementing adaptive management strategies^30^ have been suggested as a means of overcoming feedback-loop barriers that may limit seagrass recovery.

Owing to low species richness in most temperate and subtropical seagrass systems, species diversity has rarely been considered in restoration planting or monitoring. Thus, our comparison of seagrass diversity between natural and restored beds in Florida settings represents a new approach to quantifying restoration performance in the region. Natural patterns in species diversity exist within seagrass-dominated seascapes and can be dependent upon a variety of environmental drivers and disturbance histories such that a simple relationship between species diversity and age of restoration across studies may not exist. We found that the differences in seagrass diversity from restored versus natural seagrass beds were similar in all transplant and vessel damage repair projects. This was not true for restoration via sediment modification, where seagrass species diversity was lower than comparable reference beds. Because diversity was comparatively higher in older sediment modification projects, it is possible that the response was due to poor initial sediment condition. For example, inappropriate sediment grain size range, nutrient content (N, P), organic matter content, and pore water sulfide concentration, are all known to inhibit seagrass growth^31,32^. In saltmarshes, sediments restored using dredge/excavated material often exhibit slow recovery (28 + yrs.) of organic matter content, nutrient concentration, and heterotrophic activity^33,34^, and unsuitable sediment composition and cohesion, leading to sediment loss and increased turbidity, have been linked to seagrass planting failures in the past^35^. If true, it appears that sediment quality improves slowly over time.

Further, differences in seagrass community composition between restored and reference beds could be attributed to variations in life history and physiological traits among seagrass species. *Halodule wrightii* is considered a pioneering species due to its rapid branching/rhizome elongation rates, its ability to quickly colonize disturbed areas, and its wide tolerance of environmental conditions^36^, whereas *T. testudinum* is considered the climax species in the Western Atlantic and the Gulf of Mexico with comparatively slower growth rates, slower disturbance recovery rates, and narrower environmental tolerances; *S. filiforme* generally falls between the two as an intermediate successional species^24,36–38^. Restoration practitioners have exploited the pioneering traits of *H. wrightii* by planting it with the goal of reducing the successional time frame towards a climax community; i.e., compressed succession^39^. The compositional dissimilarity between restored and reference beds, commonly driven by a greater relative abundance of *H. wrightii* and lower relative abundance of *T. testudinum* in restored beds (Fig. 5A, Supplemental Table S3) comports with previous studies that have found slow rates of recovery for *T. testudinum* in seagrass restorations^24,38^. Accordingly, transplant and sediment modification projects commonly retained structural attributes characteristic of disturbed or recovering systems within the restoration timeframes examined in this study, with sparser cover and higher relative abundance of pioneering *H. wrightii*.

The variable responses of seagrass abundance in sediment modification and transplant projects may exemplify challenges associated with establishing seagrasses in un-vegetated areas where environmental conditions that initially inhibited seagrass growth are only partially abated. In contrast, vessel damage repairs achieved reference equivalency by all metrics evaluated in this study. Vessel damage repair projects inherently occur within existing seagrass beds, where there is much less uncertainty about the local environmental suitability for seagrass growth and much higher abundance of seagrass at project boundaries than other restoration type projects. Therefore, recovery is aided by lateral rhizome expansion from the surrounding meadow, so injuries resulting from propeller impacts can often recover within 5 to 10 years without further intervention^38,40^. However, impacted areas subject to high current velocities or powerful storm events may expand due to erosion unless stabilized through the use of fill material^41,42^. Vessel damage repair restorations were the most successful in supporting recovery to reference conditions in the study here and recovery mechanisms within vessel damage repair project have been the most thoroughly examined in Florida^43^; because of this, vessel damage repair remains an attractive restoration target. Nevertheless, we note that restoration via vessel damage repair may not be the preferred management prescription in all cases. For example, approaches focused on seagrass protection through education, channel markings, limited-motoring zones, and enforcement may be preferable to restoration where natural recovery is likely or ongoing vessel damage is expected^44^.

### Seagrass restoration: the co-production of knowledge

The institutional knowledge and experience gained by private-sector restoration contractors and government permitting agencies over decades appear to have fostered restoration effectiveness in Florida and may underlie the high levels of seagrass persistence recorded in our study. Unfortunately, accessing and examining data contained in monitoring reports and permitting documentation remains challenging, as gray literature is sporadically distributed across a variety of permitting agencies and a lack of standardization in data reporting standards complicates rigorous statistical inquiry. Efforts to improve public access to permitting and monitoring documentation through establishing a searchable interagency database and implementing standardized high-resolution seagrass restoration monitoring requirements would substantially expand future efforts to learn from previous and ongoing projects. These steps would help facilitate the regional synthesis necessary to advance the field of seagrass restoration during a critical time for the management of these imperiled ecosystems.

The findings from our study can be used to advance seagrass restoration and offer guidance to managers. Beyond confirming the use of restoration as a tool for seagrass ecosystem management, the compendium of past restoration projects and companion natural reference conditions demonstrate the possible suite of outcomes from seagrass restoration efforts and offer practical information on the success of different types of seagrass restoration methods and feasibility of methodology (see Table 1). For example, beyond addressing a knowledge gap, the regional differences in seagrass abundance between restored and reference beds reported in this study could serve an important role as a basis for determining areal replacement ratios for compensatory mitigation^45^. Likewise, managers could help improve our understanding of the relationship between seagrass species diversity and age of restoration by requiring the collection of information on species diversity for permitted projects. This information, along with revisits to older studies, could provide missing information and be used to improve the efficacy of monitoring program data.

More generally, the lack of standardization in monitoring and reporting has been a problematic issue in restoration ecology for decades^46^, hampering many attempts to synthesize project information^47–50^. Yet, reviews and meta-analyses are considered to be among the most important sources of information to support evidence-based decision making by restoration managers and practitioners^51^. Monitoring historic projects is an important means of addressing knowledge gaps related to the performance, function, and the long-term status of restorations^34,47,52–54^. Although identifying and reliably locating historic restoration sites can be challenging, and the cost of conducting these types of surveys can be considerable, we recommend that permitting entities and academic institutions invest in similar efforts in other regions to advance evidence-based management strategies and restoration guidelines.

## Methods

### Site selection and sampling

Seagrass restoration projects were identified through public record queries of state permitting agency databases (Florida Department of Environmental Protection, depedms.dep.state.fl.us/Oculus; South Florida Water Management District, http://my.sfwmd.gov/ePermitting), and inquiries made to environmental consulting firms directly. Criteria for site selection included: (1) the project was between 3- and 32-years post-restoration, (2) the restoration involved active habitat modification (i.e., not limited to protection measures such as signage), (3) the restored areas could be reliably located based on spatially explicit information provided in reports, and (4) seagrass was present in the project area according to the last available monitoring report. All selected projects were deemed successful or trending towards success by authors of the last available monitoring report (using a variety of criteria), thus our monitoring evaluates performance after this assessment.

Projects were grouped into three general types based on restoration techniques and environmental context. Transplant restorations involved the moving of seagrass from existing donor beds to be planted in unvegetated areas. Sediment modification restorations incorporated topographic alterations, such as filling areas too deep for seagrass recruitment or scraping down upland/submerged sediments to a depth suitable for seagrass recruitment. These projects may or may not have incorporated seagrass transplanting. Vessel damage repair restorations took place within existing seagrass beds that suffered acute injuries due to vessel activity, including propeller scars and vessel hull grounding injuries. Vessel damage restoration projects may have incorporated topographic alterations (e.g., filling with sediment or sediment in geotextile tubes), transplanting, or merely the removal of derelict vessels.

At each site, field surveys were conducted to evaluate seagrass abundance, measured in percent cover, in restored and natural reference beds. Seagrass areas nearest to restoration beds and visible in available aerial imagery were selected as reference beds. Seagrass percent cover was sampled using 1-m^2^ PVC quadrats, subdivided into 100 equally sized cells, each with an area of 100 cm^2^. Seagrass percent cover values were calculated as the number of 100-cm^2^ cells where seagrass was present within the 1-m^2^ quadrat; species-specific percent cover values were also recorded. Footprints of the seagrass restoration areas were mapped in ArcGIS (Esri) using coordinates or georeferenced maps obtained from monitoring reports. Quadrat locations were selected by random point generation within restored seagrass areas and local natural reference beds (Table 1). In some sites, such as propeller scars and other vessel damage injuries, stratified quadrat sampling along transects at regular intervals (2- to 4-m) was used to collect data in restored and adjacent reference areas outside of the injury (Supplemental Table S2).

Sampling took place in two phases; from April to June of 2016, and from August to December of 2018. Quadrat sampling points were located in the field using a handheld DGPS Trimble with ± 10-cm accuracy. Sampling effort varied between sites based on restoration area, the number of spatially distinct features associated with the restoration (e.g., vessel injuries, transplant beds, etc.), and logistical constraints—on average covering 6.6% of the restoration project area. A total of 2490 quadrat samples were taken from restored and paired reference beds at the 33 sites visited for this study, with references sampled at 60% effort of their respective restorations, on average.

### Data analysis

Univariate seagrass community structure metrics of percent cover and Inverse Simpson Diversity index (1/λ) in restored beds were evaluated relative to reference beds. Two methods were used: (1) pairwise comparisons of univariate metrics to establish the number of seagrass restoration projects that had significantly lower values than references (vote counting), and (2) the overall mean difference between paired restored and reference beds (Δµ = µ_Restored_ − µ_Reference_) to determine the effect of restoration on seagrass cover and diversity. Data obtained from quadrat samples did not meet the assumptions of parametric statistics and did not fit a known distribution, thus, non-parametric resampling-based methods were applied to raw data.

Differences in seagrass percent cover and species diversity (1/λ) between restored and reference beds were analyzed in each site using two-tailed asymptotic permutation tests with the R package ‘perm’^55^. *P*-value adjustments were applied to control for false discovery rate using the Benjamin-Hochberg procedure^56^. The effect of seagrass restoration on percent cover and diversity in comparison to references, overall and within restoration types, was analyzed with stratified bootstrap tests on paired mean differences with resampling constrained within site to account for site-specific dependencies (9,999 resamples)^57^. Bias-corrected and accelerated bootstrap confidence intervals^58^ for values of paired mean differences were generated using the ‘boot’ R package^59^. Bootstrap confidence intervals of values for paired mean differences that did not include zero were considered significantly different. Percent change values of univariate metrics are also reported as a measure of ratio-based effect size between restored and reference site mean values (Δ% = [Δµ/µ_Reference_] • 100%); to avoid ambiguity, mean difference values of seagrass percent cover are expressed in percentage points (pp). All analyses were conducted using the R statistical programming environment^60^.

Seagrass meadows in coastal Florida are composed of several species, so comparison of seagrass community composition in restored compared to reference beds was also of interest. Species relative abundance data (percent cover) were square-root transformed and used to create a Bray-Curtis dissimilarity matrix for multivariate analysis. A two-way permutational multivariate analysis of variance test (PERMANOVA; 9,999 permutations), with permutations stratified by site, was used to evaluate the effect of bed type (restored/reference) and restoration type (sediment modification/transplant/vessel damage repair) on community composition^61^. Significant interactions were followed up with pairwise comparisons of bed type within each restoration type. Community compositional dissimilarity within each restoration type was visualized using non-metric multidimensional scaling (nMDS) plots based on site mean values. Similarity percentage analysis (SIMPER) was used to identify the contribution of individual seagrass species to the overall Bray-Curtis dissimilarity between restored and reference beds^62^. Empty samples and sites that contained no seagrass in restored beds (i.e. AH, AI, H, M; Table 1) were omitted from multivariate and diversity analysis. Multivariate seagrass community structure data were analyzed using the R package ‘vegan’^63^.

The influence of restoration age on mean difference values in seagrass percent cover and diversity between paired restored and reference beds within sites were evaluated for each restoration type using linear regression. Sites that contained no seagrass in restored beds were omitted from this analysis. Restoration efforts took place in three phases over 4 years in site E; these features were treated as independent sites for temporal analysis.

The design of projects at some sites required modified analytical procedures. Project K was treated as two sites (K1, K2) to separate transplant and sediment modification features that were implemented under the same permit. An adjacent natural reference could not be identified for site AE, and so natural reference data from a local site within the same bay (AC) was substituted. A local reference was not identified for site Q, and so data from the two nearest sites, AJ and S, were pooled and substituted. No seagrass was found in restored or reference beds in sites AK and R, apparently due to system-wide seagrass loss; these sites were omitted from paired comparative analysis. Means are given with ±1 standard error unless specified otherwise.

## Supplementary information

Supplementary Information

## Data Availability

All data generated or analyzed during this study are included in this published article (and its Supplementary Information Files).
