# Supplementary material for: Long-term performance of seagrass restoration projects in Florida, USA
Source: Sci Rep. 2019 Oct 29;9:15514. doi: 10.1038/s41598-019-51856-9 (PMC6820728; doi:10.1038/s41598-019-51856-9)

Supplementary Information

Long term performance of seagrass restoration projects in Florida, USA

Ryan J. Rezek[1]; Bradley T. Furman[2]; Robin P. Jung[2]; Margaret O. Hall[2]; Susan S. Bell[1]*

[1] Department of Integrative Biology, University of South Florida, 4202 E Fowler Ave, Tampa, FL 33620, USA

[2] Fish and Wildlife Research Institute, Florida Fish and Wildlife Conservation Commission, 100 8th Ave SE, St. Petersburg, FL 33701, USA

*Corresponding author: sbell@usf.edu

Table S1. Seagrass restoration site ID, project permit number (agency)/source, and approximate project location. Agency abbreviations; FDEP = Florida Department of Environmental Protection; SFWMD = South Florida Water Management District; FDER = Florida Department of Environmental Regulation.

ID	Project Name	Permit (agency)/source	Lat (°)	Long (°)
A	Hernando Beach Channel Dredge	27-0232733 (FDEP)	28.5046	-82.6838
AA	Channel Dredge	05-264486 (FDEP)	27.8498	-80.4606
AB	Harbour Isle Development Project	56-01689 (SFWMD)	27.4557	-80.3029
AC	Bay Tree Boardwalk & Pier	56-01080 (SFWMD)	27.2934	-80.2496
AD	Pitchford's Plat Residential Boathouse	43-0262743 (FDEP)	27.2934	-80.2496
AE	Jenson Beach Boat Ramp	43-0261209 (FDEP)	27.2301	-80.2157
AF	The Reef Development	43-00680 (SFWMD)	27.1607	-80.1791
AG	Mariners Key Dock	50-07841 (SFWMD)	26.7814	-80.0455
AH	FDOT Mitigation - Snook Islands Project	50-04766 (SFWMD)	26.6264	-80.0456
AI	Bryant Park	50-09527 (SFWMD)	26.6147	-80.0459
AJ	Two Islands Marina Dredge	13-0143290 (FDEP)	25.9462	-80.1319
AK	Miami Harbor Federal Channel Expansion	0305721 (FDEP)	25.8137	-80.1577
B	SR 60 Improvements	43-00920 (SFWMD)	27.9667	-82.5563
C	North Runway Safety Area	43-01557 (SFWMD)	27.8994	-82.6446
D	Tampa Bayside Marina Expansion	29-0177106 (SFWMD)	27.8855	-82.5326
E	MacDill Seagrass Transplant Project	29-0256820 (FDEP)	27.8193	-82.5030
F	Experimental Longshore Bars	29-0268608 (FDEP)	27.8188	-82.4895
G	Treasure Island Causeway Bascule Bridge	43-027197 (SFWMD)	27.7875	-82.7700
H	Bayboro Harbor Docks	Seagrass Recovery, Inc.[a]	27.7555	-82.6274
I	Seagrass Planting Experimentation	52-411343473 (FDER)	27.7542	-82.6287
J	Shell Key	Bell et al. 2008[b]	27.6686	-82.7350
K1	Port Manatee Seagrass Mitigation	0129291 (FDEP)	27.6362	-82.5631
K2	Port Manatee Seagrass Mitigation	0129291 (FDEP)	27.6370	-82.5599
L	Canal and Bay Access Dredge	58-01637883 (FDEP)	27.4449	-82.6808
M	Coral Creek Bridge Replacement	43-027734 (SWFWMD)	26.8344	-82.2654
N	Surface Water Management	44-023887 (SWFWMD)	26.7813	-82.1646
O	Realignment of Existing Channel	36-0150335 (FDEP)	26.5528	-82.1819
P	Blind Pass Maintenance Dredge	0265943 (FDEP)	26.4869	-82.1774
Q	Florida Keys Seagrass Restoration	Mangrove Systems, Inc.[c]	25.1687	-80.3827
R	Knight's Key Bank prop scar restoration	44-0291876 (FDEP)	24.7111	-81.1278
S	Harris Gap Channel Bridge, Sugarloaf Key	44-00298 (SWFMD)	24.6552	-81.3026
T	North Roosevelt Boulevard Improvements	44-00446 (SWFMD)	24.5612	-81.7488
U	Smathers Beach	0129031 (FDEP)	24.5581	-81.7567

a - Seagrass Recovery Inc. (2003) United States Coast Guard Dock Expansion: Time-Zero Report for Seagrass Mitigation. St. Petersburg, FL.

b – Bell, S.S. et al. Evaluation of seagrass planting and monitoring techniques: implications for assessing restoration success and habitat equivalency. *Restor. Ecol.* **16**, 407–416 (2008)

c - Mangrove Systems, Inc. (1985) Combined Final Report Florida Keys Seagrass Restoration Project. Prepared for Florida Dept. of Environmental Regulation (Contract SP-73, SP-86). Tampa, FL.

Table S2. Seagrass restoration site ID, mean seagrass percent cover for restored/reference beds, standard error, sample size (*n*), percent of samples with no seagrass (Empty), and the ratio of restored and reference mean percent cover values (μ_{rest}/μ_{ref}). *S*=Stratified transect sampling, all others sampled randomly.

	Restored				Reference				
**Site ID**	**Mean cover (%)**	**SE**	***n***	**Empty (%)**	**Mean cover (%)**	**SE**	***n***	**Empty (%)**	**Mean ratio**
**A**	21.9	4.7	50	28.0	90.4	4.5	36	5.6	24.3%
**AA**	24.5	3.0	62	27.4	32.9	4.4	30	6.7	74.5%
**AB**	1.4	0.6	40	80.0	19.2	6.9	20	55.0	7.0%
**AC_S**	1.5	1.4	12	83.3	15.1	6.4	12	50.0	9.9%
**AD_S**	35.6	3.6	12	0.0	27.7	3.9	12	0.0	128.6%
**AE**	13.0	4.6	20	55.0	27.7	3.9	12	0.0	47.0%
**AF**	22.4	11.9	10	70.0	99.8	0.2	5	0.0	22.4%
**AG_S**	17.7	2.8	83	42.2	18.4	2.5	92	38.0	95.7%
**AH**	0.0	0.0	120	100.0	0.0	0.0	60	100.0	-
**AI**	0.0	0.0	40	100.0	0.0	0.0	20	100.0	-
**AJ**	78.9	4.4	30	0.0	55.5	7.5	15	0.0	142.1%
**AK**	8.0	1.8	80	50.0	75.5	4.7	40	0.0	10.6%
**B**	40.0	5.7	30	3.3	79.1	6.5	15	0.0	50.6%
**C**	73.6	5.2	60	10.0	71.1	9.9	13	0.0	103.6%
**D**	73.0	8.8	16	0.0	90.4	4.7	8	0.0	80.8%
**E**	78.2	4.1	90	4.4	97.7	2.3	44	2.3	80.1%
**F**	2.3	0.7	20	55.0	70.2	10.0	20	20.0	3.2%
**G_S**	94.2	2.1	75	0.0	99.0	0.8	39	0.0	95.1%
**H_S**	0.0	0.0	20	100.0	99.9	0.2	20	0.0	0.0%
**I**	50.1	8.7	30	43.3	99.8	0.2	20	0.0	50.2%
**J**	59.2	8.5	30	33.3	85.8	5.4	30	3.3	69.0%
**K1**	53.1	4.9	100	43.0	88.6	4.0	48	4.2	59.9%
**K2**	69.7	5.3	59	8.5	88.6	5.0	32	6.3	78.7%
**L**	52.1	4.9	66	22.7	95.3	1.2	33	0.0	54.6%
**M**	0.0	0.0	31	100.0	84.5	5.9	15	0.0	0.0%
**N_S**	69.5	4.2	65	3.1	69.6	5.2	44	4.5	99.8%
**O**	19.9	9.3	16	75.0	98.3	1.2	8	0.0	20.2%
**P**	18.9	3.8	62	38.7	15.0	5.3	30	66.7	126.2%
**Q**	81.7	2.4	150	0.7	84.0	4.1	43	0.0	97.3%
**R_S**	98.6	0.4	57	0.0	99.3	0.3	28	0.0	99.3%
**S**	19.4	7.2	30	76.7	99.7	0.3	15	0.0	19.4%
**T**	93.2	3.2	40	2.5	86.8	5.6	20	0.0	107.3%
**U**	84.1	4.6	40	0.0	92.9	3.3	20	0.0	90.5%

Table S3. Seagrass restoration site ID, mean species number m^{-2} (sn), mean inverse Simpson diversity index (1/λ), and mean percent cover values by seagrass species for restored/reference beds. Bray- Curtis similarity (%) between restored and reference beds based upon square root transformed relative abundance data (Sim) is reported. Species abbreviations: *Halodule wrightii* = *Hw*; *Thalassia testudinum* = *Tt*; *Syringodium filiforme* = *Sf*; *Ruppia maritima* = *Rm*; *Halophila decipiens* = *Hd*; *Halophila johnsonii* = *Hj*.

	Restored								Reference								
**ID**	**sn**	**1/λ**	***Tt***	***Hw***	***Sf***	***Hd***	***Hj***	***Rm***	**sn**	**1/λ**	***Tt***	***Hw***	***Sf***	***Hd***	***Hj***	***Rm***	**Sim**
A	1.42	1.22	12.0	13.2	0.0	0.0	0.0	0.0	1.32	1.16	84.6	15.5	0.0	0.0	0.0	0.0	62
AA	1.29	1.12	0.0	21.5	0.0	0.0	3.7	0.0	1.18	1.08	0.0	25.3	0.0	0.0	8.0	0.0	80
AB	1.38	1.17	0.0	<0.1	0.4	1.0	0.0	0.0	1.11	1.02	0.0	0.0	17.9	1.4	0.0	0.0	26
AC	1.00	1.00	0.0	1.5	0.0	0.0	0.0	0.0	1.17	1.04	0.0	15.0	0.1	0.0	0.0	0.0	97
AD	1.92	1.62	0.0	17.9	20.1	0.0	0.0	0.0	1.58	1.33	0.0	9.4	20.0	0.0	0.0	0.0	75
AE	1.11	1.02	0.0	6.6	0.0	3.4	3.2	0.0	1.58	1.33	0.0	9.4	20.0	0.0	0.0	0.0	17
AF	1.33	1.31	0.0	6.6	0.0	0.0	22.4	0.0	2.00	1.84	0.0	34.0	0.0	22.2	99.6	0.0	71
AG	1.06	1.01	0.0	0.0	0.0	16.9	1.1	0.0	1.11	1.04	0.0	0.0	0.0	15.3	3.4	0.0	78
AH	-	-	0.0	0.0	0.0	0.0	0.0	0.0	-	-	0.0	0.0	0.0	0.0	0.0	0.0	-
AI	-	-	0.0	0.0	0.0	0.0	0.0	0.0	-	-	0.0	0.0	0.0	0.0	0.0	0.0	-
AJ	1.93	1.70	0.0	54.1	0.0	46.6	0.0	0.0	1.93	1.72	0.0	32.1	0.0	34.8	0.0	0.0	84
AK	1.03	1.01	0.0	<0.1	8.0	0.0	0.0	0.0	2.00	1.54	13.2	52.6	30.4	0.0	0.0	0.0	38
B	1.07	1.03	2.4	0.0	0.0	0.0	0.0	37.7	1.33	1.18	8.4	0.0	0.0	0.0	0.0	74.8	10
C	1.00	1.00	0.0	0.0	0.0	0.0	0.0	73.6	1.00	1.00	0.0	0.0	0.0	0.0	0.0	71.1	100
D	1.25	1.13	1.9	0.0	0.0	0.0	0.0	71.1	1.13	1.02	0.8	0.0	0.0	0.0	0.0	90.1	89
E	1.40	1.25	13.2	3.5	74.5	0.0	0.0	0.0	1.02	1.02	34.1	0.0	65.9	0.0	0.0	0.0	57
F	1.00	1.00	0.0	2.2	0.0	0.0	0.0	0.0	1.13	1.07	5.0	20.5	54.8	0.0	0.0	0.0	27
G	1.88	1.76	79.7	74.2	0.0	0.0	0.0	0.0	1.92	1.82	86.2	79.5	0.0	0.0	0.0	0.0	86
H	-	-	0.0	0.0	0.0	0.0	0.0	0.0	1.80	1.42	65.1	17.7	49.2	0.0	0.0	0.0	-
I	1.53	1.24	21.7	30.5	3.0	0.0	0.0	3.7	1.45	1.24	33.3	5.9	75.6	0.0	0.0	0.0	22
J	1.00	1.00	0.0	59.2	0.0	0.0	0.0	0.0	1.00	1.00	0.0	85.8	0.0	0.0	0.0	0.0	100
K1	1.54	1.33	30.4	22.7	14.4	0.0	0.0	0.0	1.39	1.20	57.9	14.5	30.5	0.0	0.0	0.0	42
K2	1.11	1.04	14.9	56.4	0.0	0.0	0.0	0.0	1.33	1.20	28.3	70.9	3.1	0.0	0.0	0.0	44
L	1.47	1.31	21.1	37.2	4.1	0.0	0.0	0.0	1.06	1.03	66.1	7.8	23.5	0.0	0.0	0.0	26
M	-	-	0.0	0.0	0.0	0.0	0.0	0.0	1.00	1.00	0.0	74.2	10.3	0.0	0.0	0.0	-
N	1.11	1.07	67.5	3.4	0.0	0.0	0.0	0.0	1.05	1.03	67.5	3.0	0.0	0.0	0.0	0.0	92
O	1.50	1.26	6.3	3.1	13.6	0.0	0.0	0.0	2.25	2.05	97.5	8.8	70.3	0.0	0.0	0.0	53
P	1.00	1.00	0.0	18.9	0.0	0.0	0.0	0.0	1.00	1.00	0.0	15.0	0.0	0.0	0.0	0.0	100
Q	2.01	1.59	32.1	59.3	0.0	0.0	0.0	15.5	1.42	1.30	64.6	11.5	3.8	12.1	0.0	0.0	40
R	1.09	1.07	94.9	6.0	1.3	0.0	0.0	0.0	1.14	1.08	99.3	0.4	5.9	0.0	0.0	0.0	91
S	1.71	1.63	0.0	14.1	15.6	0.0	0.0	0.0	2.60	1.85	21.6	95.7	28.9	0.0	0.0	0.0	63
T	1.41	1.18	11.8	89.4	0.3	0.0	0.0	0.0	2.10	1.63	13.8	69.4	29.3	0.0	0.0	0.0	64
U	1.25	1.09	4.4	83.3	0.0	0.0	0.0	0.0	1.85	1.36	89.1	29.0	0.0	0.0	0.0	0.0	37

Figure S1. Results of linear regression on the influence of restoration age on site mean difference values in seagrass percent cover between restored and reference beds ($\Delta\mu = \mu_{Restored} - \mu_{Reference}$) for sediment modification, transplant, and vessel damage repair restorations.

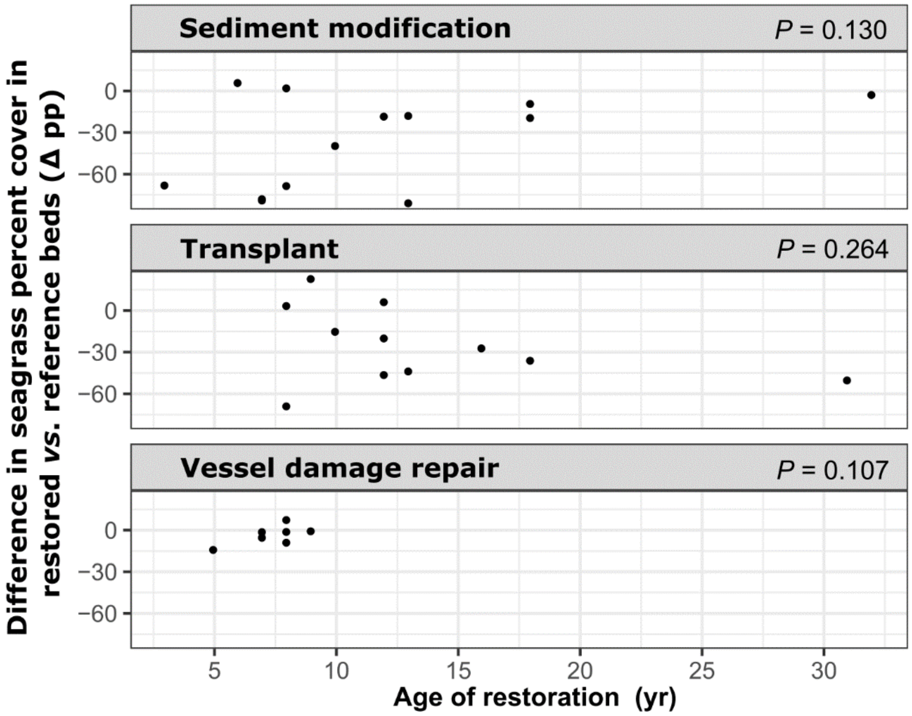

Figure S2. Results of linear regression on the influence of restoration age on site mean difference values in inverse Simpson diversity (1/λ) between restored and reference beds ($\Delta\mu = \mu_{Restored} - \mu_{Reference}$) for sediment modification, transplant, and vessel damage repair restorations. Grey shaded region indicates 95 % confidence intervals; significant P-values are in bold (α=0.05).

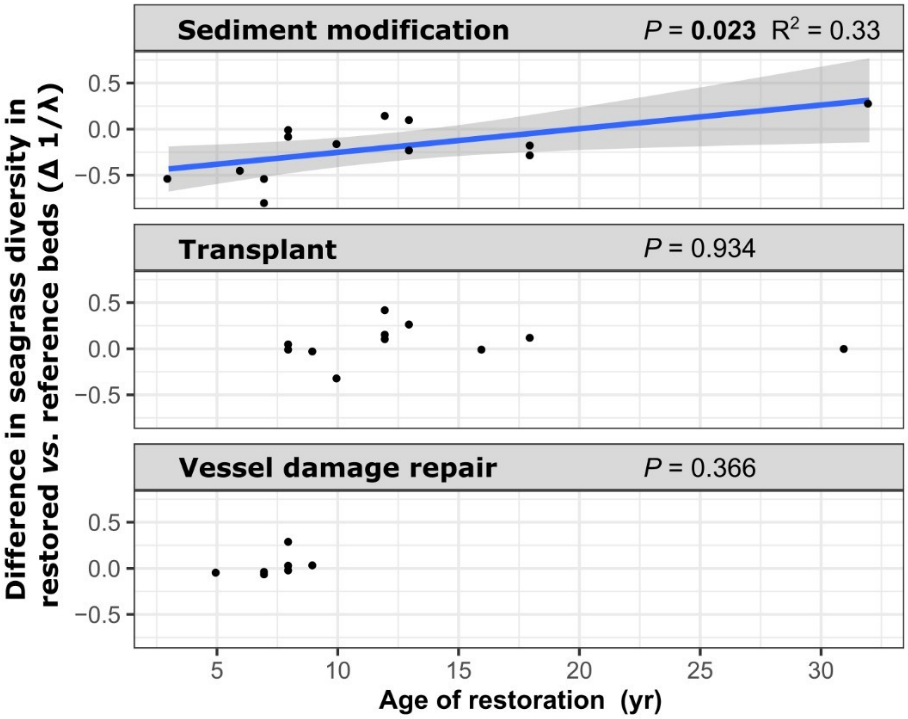

Supplement: Supplementary file 1 — Supplementary Information [file 41598_2019_51856_MOESM1_ESM.pdf]
